# Effect of Salvianolic Acid b and Paeonol on Blood Lipid Metabolism and Hemorrheology in Myocardial Ischemia Rabbits Induced by Pituitruin

**DOI:** 10.3390/ijms11103696

**Published:** 2010-09-28

**Authors:** Qian Yang, Siwang Wang, Yanhua Xie, Jianbo Wang, Hua Li, Xuanxuan Zhou, Wenbo Liu

**Affiliations:** 1 Institute of Materia Medica, School of pharmacy; Fourth Military Medical University, Xi’an, Shaanxi, 7l0032, China; E-Mails: yanqian379@163.com (Q.Y.); xieyanh@fmmu.edu.cn (Y.X.); wangjp@fmmu.edu.cn (J.W.); 561641505@qq.com (H.L.); yunyaorao@sina.com (X.Z.); 2 Department of Neurosurgery, XiJing Hospital, Fourth Military Medical University, Xi’an, Shaanxi, 7l0032, China; E-Mail: boboliucn@126.com (W.L.)

**Keywords:** coronary disease, hemorrheology, lipid peroxidation, salvianolic acid b

## Abstract

The purpose of this study was to determine the therapeutic effect of salvianolic acid b and paeonol on coronary disease. The ischemia myocardial animal model is induced by administering pituitrin (20 μg·kg^−1^) intravenously via the abdominal vein. A combination of salvianolic acid b and paeonol (CSAP) (5, 10 and 15 mg/kg BW) was administrated to experimental rabbits. Biochemical indices were evaluated during six weeks of intervention. We found that the compound of salvianolic acid b and paeonol (5, 10 and 15 mg/kg BW) can markedly and dose-dependently reduce fibrinogen and malonaldehyde levels, increase the HDL level, improve blood viscosity and plasma viscosity in rabbits. In addition, the medicine can still reduce the ratio of NO/ET and the contents of lactate dehydrogenase (LDH) and creatine phosphokinase (CPK) in a dose-dependent manner. This study demonstrates that compound of salvianolic acid b and paeonol (5, 10 and 15 mg/kg BW) can improve the blood hemorrheology, decrease oxidative injury and repair the function of blood vessel endothelium, and subsequently prevent the development of Coronary disease.

## 1. Introduction

Coronary disease (or coronary heart disease) refers to the failure of coronary circulation to supply adequate circulation to cardiac muscle and surrounding tissue [1]. Disease develops when a combination of fatty material, calcium, and scar tissue (plaque) builds up in the arteries that supply the heart with blood. Through these arteries, called the coronary arteries, the heart muscle (myocardium) gets the oxygen and other nutrients it needs to pump blood [2].

In Chinese medicine, coronary disease belongs to “chest bi syndrome and heart pain”. There are lots of prescriptions for curing “chest bi syndrome” in books of traditional Chinese medicine from past dynasties [3]. Salvianic acid A and tea catechins have been shown to have neuroprotective actions [4–6]. Many experiments have demonstrated that Sal B might be able to inhibit platelet aggregation, prevent oxidation of low-density lipoprotein, possess strong antioxidant action in rats, and reduce the initimal thickness in the air-injured carotid artery [7–10].

A complex of salvianolic acid b and paeonol is a sustained release tablets produced in our institute. This medicine is mainly composed of salvianolic acid b (content > 98%) and paeonol. In this study, we investigate the effect of this compound of salvianolic acid b and paeonol (5, 10 and 15 mg/kg BW) on lipid metabolism and hemorrheology in myocardial ischemia rabbits.

## 2. Materials and Method

### 2.1. Reagents

Salvianolic acid b and paeonol were extracted and combined (5:1) in our institute (Xi’an, China). Pituitrin injection (No. 081202) was purchased from Shanghai The First Biochemical Pharmaceutical Co. Ltd (Shanghai, China). Compound salvia pellet (No. 20080712) was purchased from Tianjin TASLY Pharmaceutical Co. Ltd (Tianjin, China). Other compounds were purchased from China and were analytical grade.

### 2.2. Animal Grouping and Treating

Rabbits (body weight 1.0–1.5 kg) (age 4 months) were provided from the laboratory animals center of the fourth military medical university (Xi’an, China) and housed individually in a room suited for the care of experimental animals. Rabbits had free access to water and were adapted to the commercial pellet feed for a week, and then were separated into the following nine groups (n = 6) by randomized block design method: a control group; a HF (Model 1) control group; a pituitrin-treated (Model 2) control group; three CSAP-treated (salvianolic acid b + paeonol) groups; a SAB-treated (salvianolic acid b) group; a paeonol-treated group or a CSP-treated (compound salvia pellet) group. The normal and model 2 control groups were fed a standard chow for 12 weeks; the model 1 control group was fed a high-fat diet for 12 weeks; the three CSAP-treated groups were fed with a high-fat diet and received the compound of salvianolic acid b and paeonol (5, 10 and 15 mg/kg BW) by gavage for 12 weeks; the SAB-treated group was fed a high-fat diet and received SAB (12.5 mg/kg BW) by gavage for 12 weeks; the Paeonol-treated group was fed a high-fat diet and received paeonol (2.5 mg/kg BW) by gavage for 12 weeks; and the compound salvia pellet (CAP) group (served as a positive control) were fed a high-fat diet and received compound salvia pellet (20 μg·kg^−1^) by gavage for 12 weeks.

After 12 weeks of medicine treatment, all drugs-treated and the model 2 control groups were administered pituitrin (20 μg·kg^−1^) intravenously via the abdominal vein to induce myocardial ischemia. Rabbits in the control group were intravenously administered corresponding volumes of vehicle solution (saline). Cardiac function was also recorded at the same time by transthoracic echocardiography (NEMIO SSA-550A, Toshiba, Tokyo, Japan).

Rabbits were then anesthetized (pentobarbital) and blood was collected from the carotid artery. The collected blood samples were immediately centrifuged at 3000 × g, plasma separated, aliquoted into small mirocentrifuge tubes and stored at −70 °C until assayed.

### 2.3. Biochemical Analysis of Serum

Malonaldehyde (MDA), lactate dehydrogenase (LDH), NO, and creatine phosphokinase (CPK) were measured in a spectrophotometer (DU800, Beckman) using commercial kits of Biosino Bio-Technology and Science Inc. (Beijing, China). Total plasma level of endothelin (ET) was measured with monoclonal antibody based sandwich immunoassay (EIA) method.

Whole-blood viscosity at shear rates from 1 to 1000 per second was measured using an automated capillary viscometer. Plasma viscosity was measured by performing Harkness capillary viscometry at 37 °C.

Hematocrit was measured using an Autocrit centrifuge (Clay Adams, USA) from venous blood collected in ethylenediaminetetraacetic acid (EDTA) anticoagulant and stored at 4–10 °C until processing.

Fibrinogen was measured with the Swain modification of the method of Larsson, Björk and Lundberg [11].

### 2.4. Statistical Analyses

All results were expressed as mean ± SD and were analyzed by SPSS for Windows, version 11.5 (SPSS Inc, Chicago, III). A value of P less than 0.05 was considered statistically significant.

## 3. Result

### 3.1. Effect of Compound of Salvianolic Acid b and Paeonol Treatment on Electrocardiogram T Wave of Rabbits

As shown in Table 1, the diagnostic value of electrocardiographic T Wave in the model groups was significantly (p < 0.01) higher than that in normal control rats. The medicine-treated groups had significantly (p < 0.05, p < 0.01) lower T Wave than the model (1 and 2) groups. Compared with model (1 and 2) groups, there was a significant (p < 0.05, p < 0.01) dose-dependent decrease in T Wave in CSAP-treated groups. The diagnostic value of electrocardiographic T Wave in the CSAP-treated group (15 mg/kg BW) (p < 0.05, p < 0.01) was significantly lower than those in SAB-treated and Paeonol-treated groups. There were no marked changes in T Wave between CSAP-treated and CSP-treated rats.

### 3.2. Effect of Compound of Salvianolic Acid b and Paeonol Treatment on Hemorrheology in Rabbits

As shown in Table 2, the whole-blood viscosity, plasma viscosity, hematocrit and fibrinogen level in the model (1 and 2) groups was significantly higher than that in normal control rats (p < 0.05; p < 0.01). The compound of salvianolic acid b and paeonol treatment (10 and 15 mg/kg BW) significantly (p < 0.05; P < 0.01) and dose dependently prevented this increase in whole-blood viscosity, plasma viscosity, hematocrit and fibrinogen level in CSAP-treated groups compared to model (1 and 2) groups. Whole-blood viscosity, plasma viscosity, hematocrit and fibrinogen level in the CSAP-treated group (15 mg/kg BW) (p < 0.05, p < 0.01) was significantly lower than those in the SAB-treated and Paeonol-treated groups. Likewise, administering 20 μg·kg^−1^ of Compound salvia pellet in CSP-treated rats significantly reduced the whole-blood viscosity, plasma viscosity, hematocrit and fibrinogen level (p < 0.01).

### 3.3. Effect of Compound of Salvianolic Acid b and Paeonol Treatment on Blood MDA, NO, ET, NO/ET, LDH, and CPK

As shown in Table 3 and 4, blood MDA, NO, LDH, CPK levels and NO/ET in model (1 and 2) groups were significantly (p < 0.05; p < 0.01) higher than those in normal control rats. Compared with model (1 and 2) controls, administration of compound of salvianolic acid b and paeonol at 5, 10 and 20 mg/kg bw significantly decreased blood MDA, NO, ET, LDH, CPK levels and NO/ET in CSAP-treated rats. Blood MDA, NO, LDH, CPK levels and NO/ET in the CSAP-treated group (15 mg/kg BW) (p < 0.05, p < 0.01) was significantly lower than those in SAB-treated and Paeonol-treated groups. As shown in Table 3 and 4, administering 20 μg·kg^−1^ of Compound salvia pellet, used as a positive control, in CSP-treated rats significantly decreased blood MDA, NO, ET, LDH, CPK levels and NO/ET.

## 4. Discussion

Pituitrin is hormones released from the posterior lobe of the pituitary, including vasopressin (antidiuretic hormone) and oxytocin. They are formed in the neuronal cells of the hypothalamic nuclei and stored in nerve cell endings in the posterior pituitary (neurohypophysis) [12]. In this study, intravenous administration of 20 μg·kg^−1^ pituitrin caused bradycardia, decreased cardiac output and induced acute myocardial ischemia. Cmpound of salvianolic acid b and paeonol treatment could alleviate pituitrin-induced acute myocardial ischemia in rabbits. A previous study showed that administration of salvianolic acid A for a period of eight days significantly attenuated isoproterenol-induced cardiac dysfunction and myocardial injury and improved mitochondrial respiratory function [13]. This was in agreement with the result of our study.

Hemorheological parameters are primary risk factors in coronary heart disease (CHD). Clinical experiments proved that whole blood viscosity and plasma viscosity were higher in patients with coronary heart disease [14]. In the present experiment, compound of salvianolic acid b and paeonol treatment could significantly reduce whole blood viscosity and plasma viscosity and improve hemorheology in rabbits.

Free radical-mediated injury has been proposed to be one of the major components involved in the pathophysiological alterations observed during ischemia and reperfusion. In the heart, reactive oxygen intermediates such as the superoxide anion and the hydroxyl radical, which are formed during reperfusion or reoxygenation of the ischemic tissue, may be responsible for the induction of electrophysiological and biochemical disturbances [15–18]. These alterations can lead to the development of reperfusion arrhythmia, myocardial stunning, or necrosis acceleration [19]. Lipid peroxidation has been hypothesized to be a major mechanism of free radicals cell damage. It may alter intrinsic membrane properties, due to physicochemical changes of oxidized lipids, or secondary to crosslinking and polymerization of membrane components affected by MDA. Lipid peroxidation may also indirectly contribute to other deleterious effects of ischemia/reperfusion, because it enhances phospholipid susceptibility to degradation by phospholipases and increases membrane calcium permeability [20–22]. Tian *et al.* [23] showed that SMND-309 (a new derivate of salvianolic acid B) decreased neurological deficit scores, reduced the number of dead hippocampal neuronal cells in accordance with its depression on mitochondria swelling degree, reactive oxygen species production, improvements on mitochondria swelling, energy metabolism, membrane potential level and mitochondrial respiratory chain complex activities. In this study, compound of salvianolic acid b and paeonol treatment could decrease plasma MDA level, indicating that the medicine may reduce oxidative injury in myocardial ischemia rabbits.

In addition, excessive nitric oxide (NO) has also been implicated in the induction of myocardial dysfunction, apoptosis, and mitochondrial respiratory chain inhibition. As the largest reservoir for O_2_ and NO in the body, hemoglobin acts as an important mediator of the nitroso-redox balance [24, 25]. Danshensu pretreatment can effectively inhibit I/R arrhythmia, ventricular arrhythmia incidence rate, onset time and duration time of VT and VT were lower than in hypertrophy groups; Danshensu were effective in preventing hypertrophy progression in rats; serum NO content and eNOS activity were significantly enhanced toward normal range [26]. In this study, a compound of salvianolic acid b and paeonol treatment reduced blood NO level, and consequently prevented angiospasm and platelet aggregation.

Endothelin (ET) is an endothelium-derived vasoactive mediator that was discovered in 1988. ET has multiple biologic actions, it contracts vascular and nonvascular smooth muscles *in vitro*, produces a sustained and long-lasting pressor response *in vivo* and exerts a positive inotropic effect on the cardiac muscle [27]. Increased plasma ET concentrations have been reported in a variety of cardiovascular disorders. ET is supposed to play a role in the pathophysiology of acute coronary syndromes. Serum CPK and LHD activities may reflect myocardiac tissue injury.

Our study showed that compound of salvianolic acid b and paeonol treatment could decrease serum ET level, CPK and LHD activities in myocardial ischemia rabbits. This indicated that this medicine may alleviate coronary artery spasm and protect myocardial cell membrane. Moreover, compound of salvianolic acid b and paeonol treatment had a synergistic part in reducing myocardial injury induced by pituitrin.

## 5. Conclusion

In summary, the present study demonstrates for the first time that compound of salvianolic acid b and paeonol can prevent angiospasm and platelet aggregation, improve myocardial cells function and blood rheology, increase coronary blood flow and reduce oxidative injury. These results suggest that the cardiovascular benefits of compound of salvianolic acid b and paeonol be explained, at least in part, by improving blood hemorheology, decreasing serum ET and MDA level, CPK and LHD activities in myocardial cells.

## Figures and Tables

**Table 1:** Electrocardiogram T Wave variation of the different groups.

Group	n	30 s	1 min	5 min	10 min	30 min
Normal	8	020 ± 0.02	0.24 ± 0.10	0.19 ± 0.15	0.19 ± 0.22	0.03 ± 0.02
Model 1	8	0.33 ± 0.05ÄÄ	0.51 ± 0.16ÄÄ	0.56 ± 0.10ÄÄ	0.59 ± 0.20ÄÄ	0.30 ± 0.14ÄÄ
Model 2	8	0.31 ± 0.06ÄÄ	0.42 ± 0.11ÄÄ	0.42 ± 0.10ÄÄ	0.39 ± 0.17ÄÄ	0.21 ± 0.12ÄÄ
CSAP (15 mg/kg BW)	8	0.17 ± 0.11**	0.28 ± 0.14**	0.23 ± 0.11**	0.19 ± 0.13**	0.04 ± 0.04**
CSAP (10 mg/kg BW)	8	0.19 ± 0.21**	0.32 ± 0.18**	0.25 ± 0.22**	0.20 ± 0.16**	0.08 ± 0.05**
CSAP (5 mg/kg BW)	8	0.22 ± 0.18*	0.48 ± 0.46ÄÄ	0.30 ± 0.21*	0.27 ± 0.22Ä**	0.13 ± 0.16Ä**
SAB (12.5 mg/kg BW)	8	0.21 ± 0.09**	0.38 ± 0.11**	0.27 ± 0.12**	0.22 ± 0.12**	0.10 ± 0.05**
Paeonol (2.5 mg/kg BW)	8	0.22 ± 0.14**	0.38 ± 0.13**	0.26 ± 0.13**	0.21 ± 0.12**	0.09 ± 0.03**
CSP (20 μg·kg^−1^)	8	0.16 ± 0.14**	0.24 ± 0.18**	0.23 ± 0.31**	0.27 ± 0.33**	0.01 ± 0.04**

Ä: P < 0.05

ÄÄ: P < 0.01, compared with normal group;

*: P < 0.05,

**: P < 0.01, compared with model group.

**Table 2:** Variations in hemorrheology of the different groups.

Group	Whole-blood Viscosity (mpa·s)		Plasma Viscosity (mpa·s)	Hematocrit (%)	Fibrinogen (g/L)
	Low Shear Rate	High Shear Rate			
Normal	7.36 ± 1.09	3.36 ± 0.21	1.12 ± 0.04	42.56 ± 2.82	1.68 ± 1.22
Model 1	13.12 ± 2.26ÄÄ	4.50 ± 0.41ÄÄ	1.37 ± 0.15ÄÄ	46.88 ± 3.78Ä	3.81 ± 1.56Ä
Model 2	10.34 ± 2.04ÄÄ	3.91 ± 0.39ÄÄ	1.23 ± 0.14ÄÄ	44.94 ± 3.47Ä	2.98 ± 1.48Ä
CSAP (15 mg/kg BW)	9.73 ± 0.45**	3.97 ± 0.55**	1.19 ± 0.07**	40.12 ± 7.03**	2.09 ± 0.63**
CSAP (10 mg/kg BW)	9.89 ± 0.98**	4.03 ± 0.18**	1.19 ± 0.05**	41.08 ± 5.72**	2.59 ± 0.55**
CSAP (5 mg/kg BW)	10.12 ± 1.21*ÄÄ	4.15 ± 0.27*	1.21 ± 0.09**	45.37 ± 6.21	2.83 ± 1.06*
SAB (12.5 mg/kg BW)	10.01 ± 0.89**	4.06 ± 0.61**	1.20 ± 0.08**	42.73 ± 8.11**	2.68 ± 0.71**
Paeonol (2.5 mg/kg BW)	9.95 ± 0.69**	3.99±0.42**	1.19 ± 0.06**	43.37 ± 8.09**	2.72 ± 0.84*
CSP (20 μg·kg^−1^)	9.78 ± 0.53**	3.92 ± 0.67**	1.17 ± 0.06**	41.09 ± 7.13*	2.11 ± 0.66**

Ä: P < 0.05

ÄÄ: P < 0.01, compared with normal group;

*: P < 0.05,

**: P < 0.01, compared with model group.

**Table 3:** Effect of compound of salvianolic acid b and paeonol treatment on blood MDA, NO, ET levels and NO/ET in the different groups of rabbits.

Group	n	MDA (nmol·mL^−1^)	NO (μmol·mL^−1^)	ET (pg·mL^−1^)	NO/ET
Normal	8	2.25 ± 0.42	54.82 ± 17.59	335.07 ± 48.21	0.16 ± 0.05
Model 1	8	3.18 ± 0.53ÄÄ	122.93 ± 23.15ÄÄ	385.77 ± 56.21	0.32 ± 0.05ÄÄ
Model 2	8	2.62 ± 0.49Ä	107.68 ± 20.12ÄÄ	378.32 ± 49.87	0.29 ± 0.05ÄÄ
CSAP (15 mg/kg BW)	8	1.95 ± 0.23*	62.68 ± 11.12**	263.45 ± 26.31ÄÄ	0.24 ± 0.05*
CSAP (10 mg/kg BW)	8	2.42 ± 0.48*	81.08 ± 12.10ÄÄ**	317.21 ± 68.12	0.26 ± 0.08*
CSAP (5 mg/kg BW)	8	2.75 ± 0.62Ä*	104.23 ± 13.42ÄÄ*	365.41 ± 75.24	0.29 ± 0.10
SAB (12.5 mg/kg BW)	8	2.25 ± 0.21*	88.53 ± 18.04ÄÄ**	321.37 ± 59.51	0.27 ± 0.06*
Paeonol (2.5 mg/kg BW)	8	2.33 ± 0.32*	83.08 ± 15.39ÄÄ**	336.72 ± 72.48	0.26 ± 0.05*
CSP (20 μg·kg^−1^)	8	1.93 ± 0.41*	60.45 ± 15.12**	258.87 ± 65.12**	0.24 ± 0.07*

Ä: P < 0.05

ÄÄ: P < 0.01, compared with normal group;

*: P < 0.05,

**: P < 0.01, compared with model group.

**Table 4:** Effect of compound of salvianolic acid b and paeonol treatment on blood LDH and CPK in the different groups of rabbits.

Group	n	LDH	CPK
Normal	8	532 ± 125	737 ± 258
Model 1	8	779 ± 318Ä	1862 ± 437ÄÄ
Model 2	8	713 ± 285Ä	1659 ± 401ÄÄ
CSAP (15 mg/kg BW)	8	564 ± 151*	790 ± 593**
CSAP (10 mg/kg BW)	8	688 ± 171	1022 ± 469*
CSAP (5 mg/kg BW)	8	763 ± 140	1715 ± 586
SAB (12.5 mg/kg BW)	8	698±173	1274±638*
Paeonol (2.5 mg/kg BW)	8	709±183	1308±516*
CSP (20 μg·kg^−1^)	8	551 ± 124	803 ± 498**

Ä: P < 0.05

ÄÄ: P < 0.01, compared with normal group;

*: P < 0.05,

**: P < 0.01, compared with model group.

## Abbreviations

CSAP: compound of salvianolic acid b and paeonol
MDA: malonaldehyde
LDH: lactate dehydrogenase
CPK: creatine phosphokinase
CAP: Compound salvia pellet
CHD: coronary heart disease
ET: endothelin
NO: nitric oxide
